# Characterization of Mechanical Property Evolution and Durability Life Prediction of Engineered Cementitious Composites Under Frozen State

**DOI:** 10.3390/ma18102375

**Published:** 2025-05-20

**Authors:** Su Lu, Liqiang Yin, Shuguang Liu, Dandan Yin, Jiaxin Liu, Huifang Hou, Lin Li

**Affiliations:** 1 Inner Mongolia Electric Power Survey & Design Institute Co., Ltd., Hohhot 010020, China; 2 School of Materials Science and Engineering, Inner Mongolia University of Technology, Hohhot 010051, China; 3 School of Resources and Environmental Engineering, Inner Mongolia University of Technology, Hohhot 010051, China; lusu0821@126.com (S.L.); liusg6011@126.com (S.L.); ljx1104@126.com (J.L.); 4 College of Water Conservancy & Hydropower Engineering, Hohai University, Nanjing 210098, China; 5 Inner Mongolia Autonomous Region Engineered Research Center for Eco-Building Materials and Fabricated Structure, Hohhot 010051, China; 6 Department of Civil and Architectural Engineering, Tennessee State University, Nashville, TN 37209, USA; lli1@tnstate.edu; 7 Department of Civil Engineering, Ordos Institute of Technology, Ordos 017000, China

**Keywords:** ECCs, freeze–thaw, low-temperature, mechanical properties, durability prediction

## Abstract

Engineered cementitious composites (ECCs) exhibit superior mechanical properties (MPs) and excellent crack control capabilities, making them widely used in practical engineering applications. However, the MPs of ECCs in frozen states (FSs), particularly their flexural properties (FPs), still need to be better understood. MP tests were designed for frozen ECC samples to investigate the service performance of ECCs in an FS. The samples underwent 0 to 300 freeze–thaw cycles (FTs), followed by compressive and flexural tests at a constant freezing temperature of −18 °C. The compressive properties (CPs) and FPs of the samples and their influencing mechanisms were analyzed. Based on this analysis, a life prediction model (LPM) for freeze–thaw-damaged (FTD) ECCs was established using the entropy weight method and the GM(1,1) model to predict the durability changes of ECCs in FS. The results indicate that with an increasing number of FTs, the uniaxial compressive strength (CS), elastic modulus (*E*), initial crack strength, and ultimate strength of ECCs in the FS are higher than those in the thawed state (TS), with a notable increase in brittleness at ultimate failure. The overall stiffness of the specimens increased under high FTs. The established model effectively predicts the durability changes of ECCs in the FS.

## 1. Introduction

Concrete is the most widely used building material on Earth, owing to its stable mechanical properties (MPs), design flexibility, and low cost [1,2]. However, this material also faces significant challenges. Concrete is inherently brittle and has low tensile strength, which, when combined with environmental factors and load stresses, leads to the inevitable cracking of structural components. Once cracking occurs, controlling the width of the cracks becomes difficult, exposing the internal structure of the material. This exposure accelerates the corrosion caused by chloride salts, sulfates, and other agents, which in turn shortens the service life of the structure [3,4]. The tendency of concrete to crack and its insufficient durability hinder the construction, expansion, renovation, and strengthening of large-scale concrete structures. In some cases, widespread cracking can even occur shortly after the project’s completion and acceptance [5], severely compromising the service safety of large infrastructure projects. Therefore, addressing the durability degradation mechanisms of conventional cementitious materials and effectively controlling the propagation of cracks during the service life of concrete structures is of paramount importance for enhancing the safety and durability of these structures.

The results of relevant studies indicate that fibers can effectively improve the toughness of concrete, reduce the formation and propagation of cracks, and enhance its durability [3,6,7]. Commonly used fibers in cement concrete include steel fibers [8], polypropylene fibers [9], glass fibers [10], and basalt fibers [11]. However, the inclusion of steel fibers introduces a relatively weak interfacial transition zone, while polypropylene fibers can decrease the workability of the mix [12]. Glass fibers have poor durability, and basalt fibers are prone to aggregation and poor dispersion, which limits their application in concrete [12]. In recent years, plant fibers have garnered widespread attention for use in cement concrete [12,13], including flax fibers [14], bamboo fibers [15], coconut shell fibers [16], and bagasse fibers [17]. Plant fibers are lightweight, renewable, and recyclable, but they face issues related to long-term effectiveness and poor durability [12,13].

Polyvinyl alcohol (PVA) fibers exhibit good affinity and durability when incorporated into cement [3,6,7,18]. Engineered cementitious composites (ECC) based on PVA can achieve up to 3% tensile strain under extreme tension, with the crack widths being controlled within 100 μm, which are considered harmless cracks [19]. Since the development of ECCs, numerous studies have focused on their preparation methods [20], MPs [21], component performance [22], and durability [3,6,7,18], yielding significant results. Research has shown that under cyclic loading, the fiber bridging between cracks provides stable and effective constraints, enhancing the structure’s damage tolerance [3,7]. Furthermore, ECCs can deform in coordination with reinforcement, avoiding issues related to stress concentration in longitudinal reinforcement and matrix spalling caused by longitudinal cracks [3]. Under high temperatures, the properties of PVA fibers undergo significant changes. At 150 °C, PVA fibers change color from yellow to orange, and at 200 °C, prolonged heating increases both peak stress and corresponding strain [23,24]. However, at 600 °C, a critical temperature is reached, corresponding to notable changes in microstructure and pore distribution, along with degradation of mechanical strength and stiffness. Moreover, when ECC specimens are heated to 600 °C, no visible cracks appear. This is due to the random distribution of PVA fibers in the matrix; as the temperature increases, cracks and water vapor gradually form at the matrix interface. The water vapor escapes through these cracks, and when the temperature exceeds the melting point of the fibers, the melted fibers leave voids, which helps release vapor pressure within the matrix, preventing spalling [25].

However, over half of the global regions are classified as cold regions, including much of Europe, northern North America, Hokkaido in Japan, Russia, and northern China [7,26]. With the growing demand for space and energy worldwide, the construction of projects in cold and harsh environments has increasingly attracted the attention of researchers [27,28]. The rapid expansion of industrial facilities or building structures in high-latitude or high-altitude areas has resulted in structures often being subjected to FTs during their service life. Li [19] and Şahmaran et al. [29,30] conducted 110 rapid freeze–thaw tests on ECC and ordinary concrete prism samples using the quick-freeze method. The ordinary concrete samples experienced severe degradation, while the ECC samples exhibited almost no reduction in relative dynamic modulus. Şahmaran et al.’s [29,30] research also indicated that the tensile strain capacity of ECC samples decreased to around 2% after FTs300. Özbay [31,32] showed that after FTs, the bending toughness of ECC samples significantly decreased, with the reduction in bending stiffness being more pronounced than the reduction in bending strength. Nam et al. [33] studied the changes in the pore characteristics of ECC samples before and after FTs 300 using mercury intrusion and linear scanning cross-sectional methods. The results indicated that after FTs 300, the number of pores larger than 100 nm in diameter in the matrix samples decreased significantly, and they concluded that the freeze–thaw resistance of ECCs can be controlled by the characteristics of the fibers and the pore structure of the cement matrix. These studies confirm the excellent freeze–thaw resistance of ECCs; however, research on the MPs and durability of ECCs under low-temperature freezing conditions is scarce in the literature. Most studies by domestic and international scholars focus on preliminary experimental and theoretical research on concrete or UHPC at low temperatures. Dahmani et al. [34] demonstrated that the compressive strength (CS) of low-temperature concrete can be two to three times higher than that at room temperature. However, the strength gain does not follow a uniform pattern with temperature changes, meaning that continued temperature reduction does not consistently improve CS. Generally, CS increases to a peak value with decreasing temperature, followed by a plateau or a drop, and the temperature corresponding to the peak CS differs significantly across concrete mixes, with moisture content being the primary influencing factor [28]. Kim and Yoo [35,36] studied the pull-out behavior of steel fibers under low-temperature conditions, pointing out that regardless of loading rate or fiber geometry, the bond strength between steel fibers and the cement matrix increases at low temperatures. Zhang et al. [8,37] found that the increased peak flexural strength of UHPC at extremely low temperatures is related to the freezing of pore water, and this freezing process may also reduce the post-peak toughness.

In summary, existing research primarily focuses on the performance of ECCs after FTs and under the combined effects of freeze–thaw and loading. Studies on the MPs of ECCs under low temperatures are relatively limited. To further promote the application of ECCs in high-latitude cold regions, it is essential to investigate the MPs of ECCs under low-temperature conditions. This study focuses on analyzing the evolution of uniaxial compression and bending performance of ECCs at low temperatures and exploring the underlying mechanisms. Additionally, an entropy-weighted method is used to establish a freeze–thaw durability (FTD) assessment model for ECCs in both the thawed and frozen states (FSs). Based on the durability values, a life prediction model for ECCs in both the thawed and FS is developed using grey theory. The model’s accuracy meets the requirements and can provide theoretical support for the use of ECCs in freeze–thaw environments in practical engineering applications.

## 2. Experimental Details

### 2.1. Raw Materials

The ECC specimens in this experiment were prepared using P·O42.5R ordinary Portland cement produced by the Jidong Cement Plant in Hohhot, China. The chemical composition of the raw materials was tested using the Shimadzu Model 1800 X-ray fluorescence spectrometer (XRF) in Tokyo, Japan, as shown in Table 1 and Table 2, which display the oxide composition and physical properties of the cement. The physical performance of the cement was tested according to the “Standard Consistency Water Requirement, Setting Time, and Stability Test Methods for Cement” (GB/T 1346-2011) [38] and the “Method for Cement Mortar Strength Testing (ISO Method)” (GB/T 17671-2021) [39]. The test results are presented in Table 2. The fly ash used was Class I high-calcium fly ash produced by the Dalate Power Plant in Ordos City, China, with its main oxide composition listed in Table 3. The quartz sand selected was 109–212 μm refined quartz sand from the Hohhot Quartz Sand Group in China. The sand’s related indicators were tested according to “Quartz Sand” (DB34/T 1056-2009) [40] and “High Purity (SiO_2_ ≥ 99.997%) Quartz Sand” (DB43/T 1167-2016) [41], with the primary indicators shown in Table 4. The water-reducing agent used was the Sika-III polycarboxylate-based water reducer, produced by Sika Company in Dalian, China, with a water reduction rate of approximately 33%. The fibers used were Kuralon K II PVA fibers, produced by Kuraray Company in Japan, with the performance indicators of the PVA fibers, including elastic modulus (*E*), elongation, and tensile strength, listed in Table 5, following the testing standards outlined in “Synthetic Fibers for Cement Concrete and Mortars” (GB/T 21120-2018) [42].

**Figure 1:**
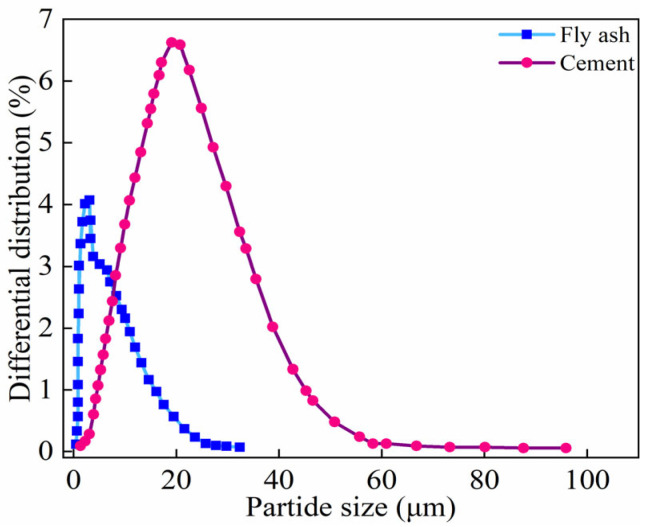
Particle size of Portland cement and FA.

The particle size of Portland cement and fly ash was tested using the WJL606 laser particle size analyzer, produced by Shanghai Instrument and Electronic Materials Optical Co., Ltd. in Songjiang District, Shanghai, China. The results are shown in Figure 1. The particle size range of fly ash is approximately 0–30 μm, with the higher variation occurring around 5 μm. The particle size range of Portland cement is approximately 0–100 μm, with the higher variation occurring around 20 μm.

### 2.2. Specimen Preparation

**Figure 2:**
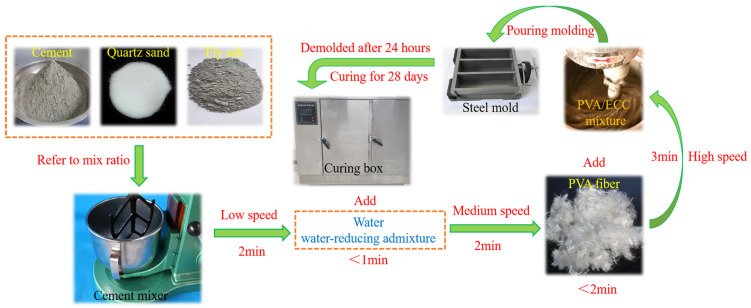
Preparation process of ECC specimens.

Table 6 displays the mix proportions of ECC specimens, while Figure 2 depicts the preparation process. The FA constitutes 80% of the total weight of the cement and FA, resulting in a mass ratio of FA to the cement of 4:1. ECC specimens maintain a water–cement ratio of 0.24, employing ordinary tap water as the mixing medium. Following the guidelines of GB/T 17671-2021 [39], the ECC mixture is cast into precast steel molds with dimensions of 40 mm × 40 mm × 160 mm, and the molds are de-molded 24 h after casting. In adherence to the prescribed standard testing procedures outlined in GB/T50081-2016 [43], we place the specimens within a standard curing chamber. In this controlled environment, we meticulously maintain a temperature of 20 °C ± 2 °C while ensuring that humidity levels remain consistently above 95%. This curing process persists for 28 d.

### 2.3. Test Method

**Figure 3:**
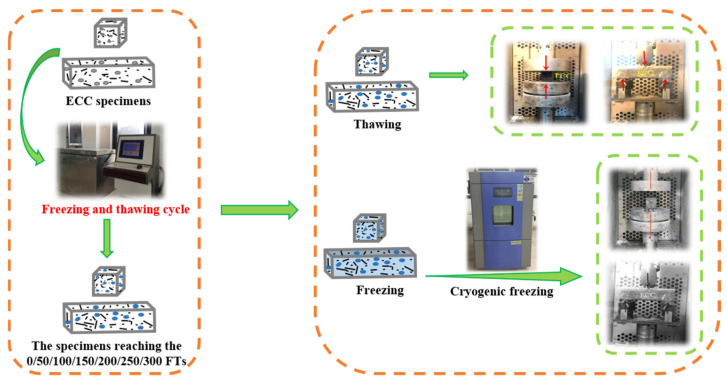
Flow chart of MP test of ECC specimens in UFS and FS.

As shown in Figure 3, the flowchart of the MP testing process for ECC specimens under TS and FS is provided. The FTs test follows the rapid freeze–thaw method in the “Standard for Test Methods of Long-Term Performance and Durability of Ordinary Concrete” (GB/T 50082-2009) [44]. The TDR-type concrete freeze–thaw testing machine, controlled by a microcomputer, is used to conduct FTs0–FTs300 on ECC prismatic samples. The temperature of the specimen is monitored by a temperature sensor embedded in the center of the sample, with a temperature range of (−18 ± 2) °C to (5 ± 2) °C. Each FT is completed within 2–4 h. The uniaxial compression test and bending test follow the standards of the Ministry of Building Materials Industry of the People’s Republic of China (JC/T 2461-2018) [45] and the Japan Society of Civil Engineers (JSCE) recommended standards. Research by Stockhausen et al. [46] indicates that pore water can freeze extensively below 0 °C, hence defining room temperature (20 °C ± 2 °C) as the TS and a sustained low temperature of −18 °C ± 2 °C as the freezing state. The MP tests are conducted on an MTS servo-hydraulic loading system using displacement control at a loading rate of 0.1 mm/min. Load and deformation are measured using sensors attached to the loading system. The CS test uses prismatic specimens with dimensions of 40 mm × 40 mm × 40 mm, and the loading rate is 0.1 mm/min. The bending performance test uses prismatic small beam specimens with dimensions of 40 mm × 40 mm × 160 mm, conducted on the MTS servo-hydraulic loading system, employing a three-point bending displacement control method with a loading rate of 0.2 mm/min and a test span of 120 mm.

In the case of FS PVA-ECC MP testing, the procedure is as follows, as depicted in Figure 3:

1. Subject the ECC specimens, which have undergone a specified number of FTs0–FTs300, to a saturation treatment. Wrap them with plastic wrap to prevent moisture loss.

2. Post-treatment, subject the ECC specimens to freezing in a low-temperature chamber. Systematically lower the chamber temperature from 5 °C to −18 °C, and hold the specimens at −18 °C for a minimum of 4 h, ensuring the thorough conversion of pore water into solid ice.

3. After the freezing treatment of ECC specimens is completed, maintain a constant temperature of −18 °C in the environmental chamber of the MTS loading system and conduct single-axis compression and flexural testing on the specimens in the FS.

### 2.4. Data Analysis Method

To evaluate the contribution of low-temperature freezing to the increase in FS of ECC specimens, an FS gain ratio is defined and calculated as follows [8,12]:

(1) $$\gamma_{\mathrm{cn}}=\frac{f_{bn}^{f}-f_{bn}^{t}}{f_{bn}^{t}}\times 100\%$$

where $\gamma_{cn}$ is the gain ratio of FS at FTs *n*, $f_{bn}^{f}$ is the ultimate FS at FT *n* in the FS, and $f_{bn}^{t}$ is the ultimate FS at FT *n* in the TS.

To assess the reliability of the experimental results, error bars are included in the bar charts to represent the standard deviation (SD) of the measured values. The inclusion of error bars is crucial for clearly displaying the distribution or variability of the data and demonstrating the consistency of measurements in repeated experiments. These error bars are calculated from three tests conducted under the same conditions to ensure the stability and repeatability of the results. For each ECC sample, the standard deviation of key performance parameters, such as initial cracking strength, ultimate bending strength, ultimate peak deflection, and bending toughness index, measured under different FTs, is calculated. The standard deviation is calculated using the following formula:

(2) $$SD=\sqrt{\frac{1}{n-1}\sum_{i=1}^{n}{\left(x_{i}-\bar{x}\right)}^{2}}$$

where *x_i_* represents each individual measurement, $\bar{x}$ is the mean value of the measurements, and *n* is the number of replicates (in this case, *n* = 3 for triplicate experiments). The error bars in the figures reflect the variability of these properties, providing a clearer understanding of the precision of the experimental results.

## 3. Test Results and Discussion

FTs include both freezing and thawing phases. Typically, when studying the effects of FTs on the MPs of concrete materials, experiments are conducted only during the thawing phase, i.e., the conventional TS, rarely involving the freezing phase. However, in engineering structures exposed to FT environments, structures subjected to loads in low-temperature FS are also common. Therefore, this chapter conducts compression/flexural tests on ECC specimens after undergoing different FTs in FS to explore the characteristics of the MP evolution of ECCs in both TS and FS during FTs. Given that the research team has previously conducted extensive studies on the compression MPs of ECCs, this study builds upon the research mentioned in references [7,26] and utilizes the relevant experimental data from their TS and FS ECC specimens for calculation. Furthermore, we conduct corresponding analyses on the FP tests of ECC specimens in both TS and FS conducted in this study.

### 3.1. Uniaxial Compression Performance

#### 3.1.1. Stress–Strain Curve

**Figure 4:**
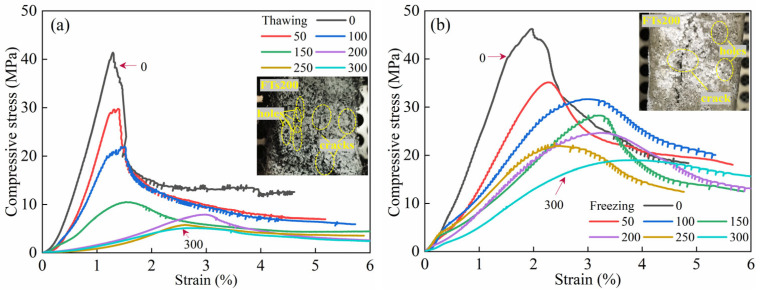
Uniaxial stress–strain curves of ECC specimens under thawing and freezing conditions [7,26]. (**a**) Thawing; (**b**) Freezing.

As shown in Figure 4a,b, the uniaxial compressive stress–strain curves of ECC specimens in both TS and FS, obtained in previous experiments by the research team [7,26], illustrate the freeze–thaw evolution characteristics. In both loading environments, the uniaxial compressive stress–strain curves of ECC at different FTs exhibit a trend of initially rising to the peak point and then gradually falling off, eventually stabilizing. The uniaxial compressive stress–strain curve of ECCs in the TS can be roughly divided into four stages based on its deformation and fracture process. The first stage is characterized by the elastic deformation of both the fibers and the matrix. In the second stage, the fibers remain elastically deformed, while the matrix deformation becomes inelastic. The third stage involves both the fibers and the matrix undergoing inelastic deformation. The fourth stage is marked by fiber fracture, followed by composite material failure. In the TS, the curve at FTs0 does not exhibit a sharp decline after reaching the peak load, as seen in typical concrete. Instead, after the “inflection point,” the curve shows a phase where deformation continues to increase while strength gradually decreases, which corresponds to the ductile failure characteristics of ECCs under ultimate compression. As the FTs increase, the peak CS of the ECC uniaxial stress–strain curve shows a significant drop. After more than FTs100, the peak CS of the stress–strain curve drops below 10 MPa. After FTs200, as the number of cycles continues to increase, there is no significant difference in the stress–strain curve except for the peak point.

The uniaxial compression stress–strain curve of ECCs in the FS is markedly different. The curve does not exhibit a distinct “inflection point”. While the curve of FTs0 after the peak shows a similar inflection point, compared to the curve in the TS, the rate of stress drops after the inflection point increases more rapidly as strain increases, presenting characteristics of brittle failure. Unlike the melting curve, where the “inflection point-like” feature is still observable after FTs100, the frozen curve does not show any further “inflection points” as the FTs increase. Although the stress–strain curve overall shows a declining trend as the number of FTs increases, the drop in stress after the peak is relatively small. After FTs300, the final strength of the stress–strain curve remains around 20 MPa.

Taking the case of FTs200 as an example, Figure 4a,b also display the uniaxial compression failure modes of ECC specimens after FTs under different environments. Figure 4a shows the compressive failure of ECC specimens in the TS, where both vertical and horizontal deformations are large, indicating clear ductile failure characteristics. However, due to significant FTD, the macroscopic surface exhibits severe honeycomb-like spalling, which reflects the serious degradation of load-bearing capacity to some extent. In contrast, the scenario shown in Figure 4b under the FS is entirely different, with relatively smaller vertical and horizontal deformations and no obvious compressive deformation, as seen in Figure 4a. From the crack perspective, ECC specimens in the low-temperature freezing environment typically exhibit only one macrocrack throughout the failure process, displaying clear brittle failure characteristics. The failure modes of ECC specimens in both environments are consistent with the failure patterns indicated by the stress–strain curves.

As shown in Table 7, the uniaxial compression MP parameters of ECC specimens in both TS and FS during the FTs0–FTs300 cycles obtained from the previous experiments of the research group [7,26] are presented.

#### 3.1.2. Peak Strength

**Figure 5:**
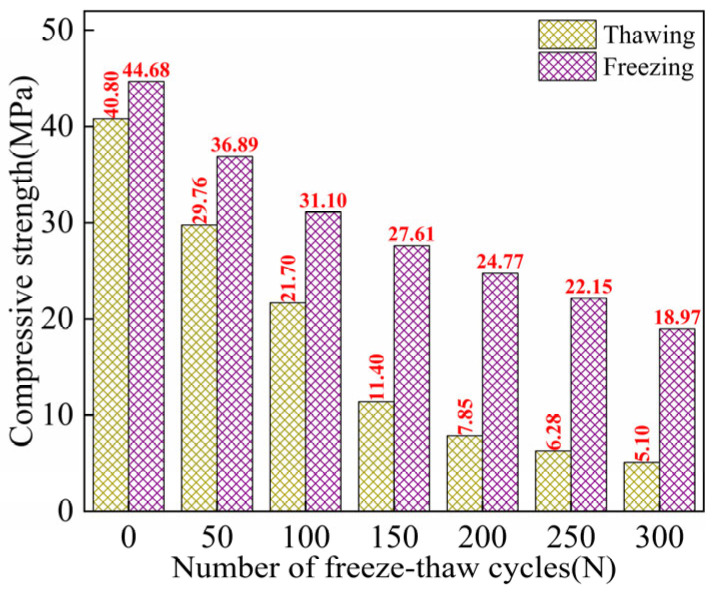
Freeze–thaw evolution characteristics of peak strength of ECC specimens in thawing and freezing states [7,26].

Figure 5 shows the freeze–thaw evolution characteristics of peak strength for ECC specimens in both TS and FS. As the number of FTs increases, the peak strength of ECC in both the TS and FS gradually decreases. At each FT, the peak strength of ECC in the freezing state is higher than that in the TS, and there is a strength difference between the freezing and melting peak strengths, which increases with the number of FTs. At FTs0, the strength difference between the two states is 3.88 MPa, and after FTs300, the strength difference increases to 13.87 MPa. Cai et al. [47] and Rong et al. [48] found that in a negative temperature environment, the CS of concrete increases as the temperature decreases. The critical value of CS occurs around −120 °C. When the temperature drops to −120 °C, the CS of concrete no longer increases. At this point, the CS of water-saturated concrete can even exceed that at room temperature by 100 MPa. Moreover, studies have shown that the CS of fiber-reinforced concrete in a freezing state is indeed higher than that at room temperature [7,26]. In the freezing state, after FTs300, the peak strength of the ECCs decreased by 25.7 MPa compared to ECCs without FTs. From FTs0–100, the peak strength dropped rapidly, decreasing by 13.6 MPa, while from FTs100–300, the rate of peak strength reduction slowed, with a decrease of 12.1 MPa.

The peak strength loss rate of ECC in the freezing state during FTs0–FTs300 is consistently lower than that of ECC in the TS. Calculations reveal that as the number of FTs increases, the peak strength loss rate of the samples in both TS and FS significantly increases. However, the rapid increase in loss rate in the TS occurs during FTs0–FTs200 cycles, with a loss rate of 80.76%. In contrast, the rapid increase in loss rate in the freezing state only occurs during the FTs0–FTs100 cycles. After FTs300, the peak strength loss rate of ECC in the freezing state is only 57.55%, much lower than the 87.51% observed in the TS.

This can be attributed to the beneficial effect of the freezing-induced expansion of pore water at negative temperatures, which generates an expansive force that acts as prestress for the ECC. The CS of cement-based materials at negative temperatures can be considered the sum of CS at room temperature and gain value at low temperatures. Research by Jiang et al. [49,50,51] suggests that the lattice-like ice structure puts the specimen into a multiaxial stress state, effectively applying prestress. When the capillary pores in concrete are completely filled, the ice can directly resist external loads, eliminating stress concentrations in the specimen and suppressing the development of microcracks. As the FTs increase, ECC FTD becomes more significant. The strength of ECCs in the TS severely degrades, whereas ECCs in the freezing state maintain their strength due to the prestress provided by the pore ice, which prevents rapid degradation. This strength contribution becomes more evident at high FT counts.

#### 3.1.3. *E*

**Figure 6:**
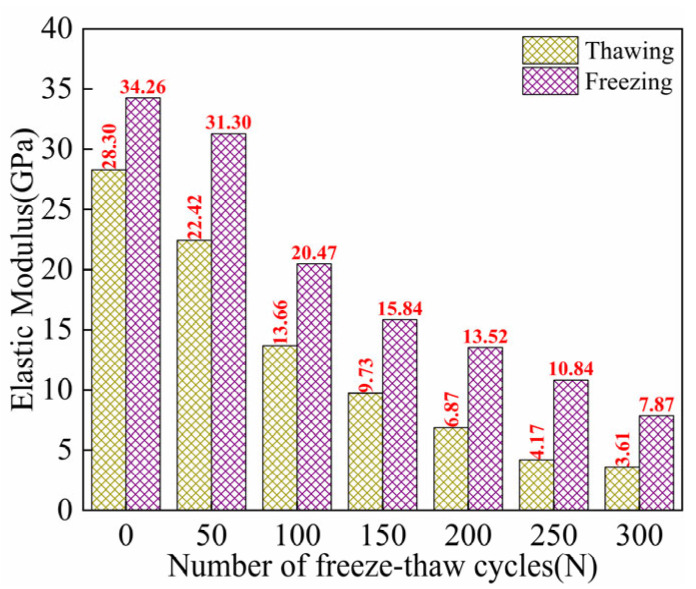
Freezing–thawing evolution characteristics of *E* of ECC specimens in thawing and freezing states [7,26].

*E* is one of the key parameters for evaluating the MP of ECC. Figure 6 illustrates the freeze–thaw evolution characteristics of the *E* of ECC specimens in both TS and FS. It can be observed that as the number of FTs increases, the *E* of ECCs in both environments gradually decreases, with a significant decline in values. Throughout each FT, the *E* of ECCs in the freezing state is much higher than that of ECCs in the TS.

Under melting loading conditions, the initial *E* of ECCs is 28.30 GPa. After FTs50, the *E* of the specimen decreases slightly by 5.88 GPa. During FTs50–FTs300, a substantial drop in the *E* is observed, with a loss rate of 87.24% compared to the initial state. In the freezing state, prior to undergoing FTs, the *E* of the specimen is 34.26 GPa. The most significant decrease in *E* occurs from FTs0 to FTs100 cycles, with a change of 13.78 MPa. After FTs300, the *E* decreases to 7.87 GPa. Throughout the entire FTs0–FTs300 FTs, the loss rate of the *E* reaches 77.03%. Compared to the TS, ECCs in the freezing state exhibit a lower loss rate of *E* throughout the entire FT.

The *E* of concrete materials changes with decreasing temperature. In the FS, the variation of the *E* of concrete depends on the amount of ice formation in the material and the extent of material damage caused by the solid ice [49,50,51]. Analysis suggests that the freezing of pore water in ECCs after low-temperature treatment leads to an increase in the overall stiffness of the material, enhancing its load-bearing capacity. As a result, the initial *E* is higher, and the loss rate of the *E* after FTs is lower. Research by Yao et al. [52] demonstrated that continuous cooling treatment of materials results in two opposing effects. One is the hardening effect caused by the formation of solid ice in the material’s pores, as the stiffness of solid pore ice is higher than that of liquid pore water. The other is the weakening effect, where excess solid ice generates high pressure on pore walls, causing material damage. When the hardening effect dominates, the overall stiffness of the material increases. Conversely, when the weakening effect dominates, the overall stiffness of the material decreases. This study corresponds to the first case, where the pore water in the material undergoes a phase change to ice at low temperatures. The hardening effect of pore ice predominates, and the damage caused by pore ice is a secondary factor. Consequently, the overall stiffness of the ECC increases, resulting in a higher initial *E* and better durability.

### 3.2. Flexural Performance

#### 3.2.1. Bending Failure Mode

**Figure 7:**
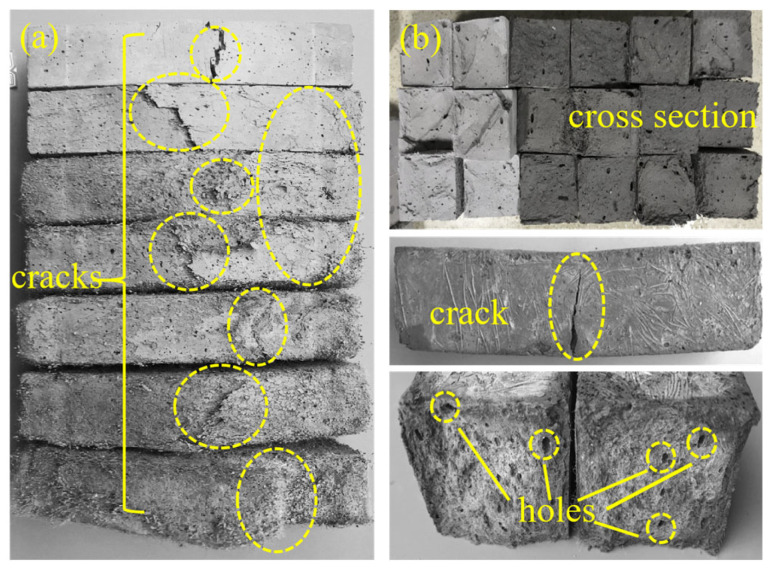
Flexural failure patterns of ECC specimens in UFS and FS. (**a**) Thawing; (**b**) Freezing.

As shown in Figure 7, the bending failure modes of ECC specimens under two loading environments are illustrated. It can be observed that throughout FTs0–FTs300, ECC specimens in the TS exhibit significant bending deflection during bending failure, showcasing a clear ductile failure characteristic. After FTs300, due to severe damage and the insufficient bearing capacity of the cross-section, the failure mode shows signs of shear failure and is accompanied by delamination. In contrast, the bending failure mode of ECC specimens in the FS is entirely different. After bending failure, the specimen breaks into two parts. Upon observing the fracture surface, it is relatively smooth, with no fibers being pulled out. Instead, all fibers are broken. From the crack perspective, only a single straight-line macrocrack appears throughout the bending failure process in the FS, with fewer cracks but larger widths. This is in stark contrast to the crack distribution in ECC specimens in the TS. This difference in crack distribution can be attributed to the freezing of liquid water at low temperatures, which provides additional bonding strength along the fracture surface [53]. As a result, the ECC specimen exhibits a brittle failure characteristic. Moreover, the frozen water reduces the porosity of the cement matrix, increasing the uniformity of the ECC and reducing the randomness of crack distribution [54,55]. The brittle bending failure mode, with all fibers being broken in the small beam sample cross-section, may contribute significantly to the bending load-bearing capacity, fundamentally reflecting the trade-off between strength and toughness.

#### 3.2.2. Load-Deflection Curve

**Figure 8:**
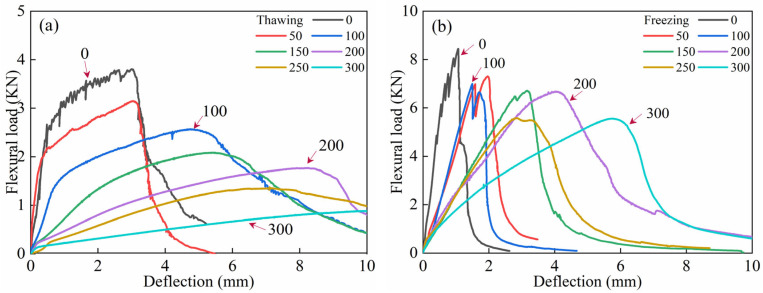
FTE characteristics of flexural load-deflection curves of ECC specimens in UFS and FS. (**a**) Thawing; (**b**) Freezing.

As shown in Figure 8, the bending load-deflection curves of ECC specimens in both TS and FS during the FTs0–FTs300 cycles exhibit the evolution characteristics of FTs. These curves provide insight into various bending MP parameters of ECCs, such as bending strength, initial cracking strength, and ultimate peak deflection. As presented in Table 8, the bending MP parameters of ECC specimens in both TS and FS are summarized for FTs0–FTs300.

As shown in Figure 8, for each FT, the load-deflection curve of ECC specimens in the FS exhibits a significantly higher ultimate peak load than in the TS. In the TS, the bending failure process of ECCs can be divided into three main stages: Elastic Deformation Stage (Stage I): From the origin to the initial crack point, the load-deflection curve shows linear elastic growth. During this stage, the ECC specimen experiences mainly elastic deformation, with a relatively small load and no crack propagation. The bonding between fibers and the matrix is good, with no slippage, and the specimen’s resistance is composed of the matrix’s cohesive force, aggregate interlocking, and the bond strength between the matrix and fibers. The stress–strain curve is approximately linear. Strain-Hardening Stage (Stage II): From the initial crack point to the ultimate peak load, as the deformation increases, the load gradually increases and is accompanied by the development of multiple cracks, with new peaks appearing in the curve. The curve shows a strain-hardening trend. During this stage, the cement matrix exits the working phase, and PVA fibers begin to perform bridging and crack resistance functions. Specifically, PVA fibers bridging across cracks transfer stress via bonding across the crack. This stage demonstrates the specimen’s good toughness, and the load mainly supports the pullout or fracture of the fibers. During crack propagation, the sound of fiber breakage may be heard. Instability and Failure Stage (Stage III): After the ultimate peak load, as deformation continues to increase, the load rapidly decreases. However, due to the bridging effect of the fibers, the specimen does not immediately fail. The curve shows a strain-softening development trend, continuing until the specimen fails. During failure, the stress gradually decreases, while strain increases, and some fine cracks may gradually close, exhibiting ductile failure characteristics. These findings align with the studies of [56]. In contrast, in the FS, ECC specimens’ load-deflection curves do not exhibit significant strain-hardening characteristics, which may even completely disappear. As the number of FTs increases, the bending load-deflection curve shows a decreasing trend, but the peak load reduction is less significant compared to the TS.

As shown in Table 8, during FTs0–FTs300, the peak load of the curve decreased by 2.89kN, and after FTs300, the peak load still remained above 5 kN, indicating that the FS PVA-ECC retains a certain flexural bearing capacity even after a high number of FTs. Comparing the rising segments of the load-deflection curves at different FTs, the slope of the curve at FTs0 is the steepest, at 9.73. The slopes at FTs50 and FTs100 are relatively similar, at 4.41 and 4.70, respectively. From FTs150 to FTs250, the slopes of the rising segments generally decrease with slight fluctuations, going from 2.33 to 1.95 and then increasing to 2.12. After FTs300, the rate of increase in the rising segment of the curve significantly slows, with the slope reducing to 0.97. This indicates that the load increase effect is less noticeable than in previous FTs, but the deflection change of the sample is more pronounced, with the deflection reaching nearly 6 mm at the peak load. Observing the descending segments of the curves at different FTs, it is noted that as the FTs increase, the post-peak development of the curve gradually becomes more gradual. The curve at FTs0 drops sharply after reaching the peak load, while the curves at FTs50–FTs150 exhibit a similar post-peak development trend. For the curves at FTs200 and beyond, the post-peak phase is characterized by a significant increase in deflection but a slower decrease in load.

It is analyzed that in the TS, due to the hydrophilic nature of PVA fibers [3,6,7,26,57], they form a strong and stable bond with the cement matrix. After loading, the main failure mode is fiber pull-out associated with the propagation of the main crack, which effectively dissipates energy. Therefore, the post-peak region of the load-deflection curve in the TS is more pronounced. The cement matrix contains chemically bound water (α-water) and physically adsorbed water (β-water), both with strong bonding forces. In the FS, the phase transition of β-water is mainly induced, while α-water remains relatively stable [49,50,51,57]. The freezing process begins in the larger pores of the cement matrix and gradually extends to the gel pores, increasing the density of the cement matrix. During this stage, the small volume expansion of ice induces microcracks in the weaker regions of the cement matrix. However, the surrounding PVA fibers can bridge these microcracks and prevent the propagation of macroscopic cracks. Furthermore, the low-temperature effect enhances the wrapping effect between the PVA fibers and the cement matrix, expanding the mechanical anchoring area of the fibers and increasing mechanical friction during the pull-out process [58]. The bonding force between ice and the cement matrix is strengthened, improving the integrity of the ECC and slowing the expansion of microcracks within the material [49,57]. As a result, in the FS, the energy dissipation capacity of ECC is significantly enhanced, requiring more energy to turn microcracks into macroscopic cracks. Therefore, the bending load-deflection curve of the ECC specimen in the FS undergoes significant changes in development, with a severe degradation or even disappearance of its strain-hardening performance. It is evident that ECC exhibits brittle failure characteristics under low-temperature conditions, a phenomenon known as the temperature-brittleness effect [59].

#### 3.2.3. Initial Cracking Strength

**Figure 9:**
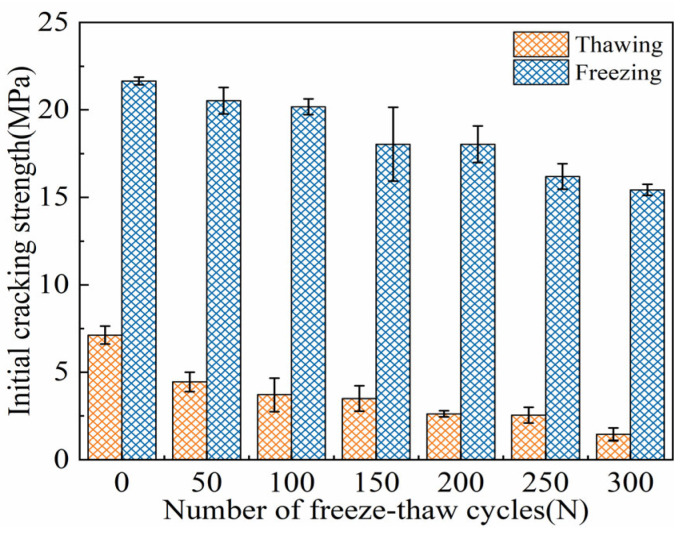
FTE characteristics of initial crack strength of ECC specimens in UFS and FS.

As shown in Figure 9, the initial cracking strength evolution characteristics of ECC specimens under both TS and FS are depicted. With the increase in the number of FTs, the initial cracking strength of the ECC specimens in both states shows an overall decreasing trend. At 0 cycles, before freeze–thaw exposure, the initial cracking strengths in the TS and FS were 7.12 MPa and 20.46 MPa, respectively. The gain in the initial cracking strength provided by the negative temperature environment reached 204.07%. After FTs300, the initial cracking strengths of the ECC specimens in both loading environments decreased to 1.44 MPa and 15.55 MPa, respectively. At each FT, the initial cracking strength of the ECC specimens in the FS was significantly higher than that in the TS. Over FTs0–FTs300, the strength difference between the two states ranged from 13.07 MPa to 15.97 MPa.

The analysis suggests that the higher initial cracking strength in the FS is due to the PVA fibers, which are randomly distributed in the matrix to form a three-dimensional network structure, contributing to the overall stability of the material [3,18]. Additionally, the freezing of pore water into ice in low-temperature conditions enhances the bond between the cement matrix and the fibers. As a result, the initial cracking in ECC specimens occurs not from fiber slip or pull-out but from fiber pull-out in certain cross-sections of the bending tensile zone. In low-temperature conditions, the PVA fibers and cement matrix exhibit good synergistic effects. The growth of ice crystals in the FS is the primary factor affecting the material’s performance. Consequently, fiber pull-out is induced by ice expansion in the micropores at the fiber/matrix interface, leading to higher interfacial bond strength [49]. In the TS, the bending test reveals that the mortar’s tensile strength and ultimate strain are quite low, and the initial cracking strength mainly depends on the load-bearing capacity of the PVA fibers. These fibers can store energy, protecting the ECC specimen from premature failure during FTs, and they provide substantial load-bearing capacity during bending tests. However, in the FS, the presence of pore ice causes a hardening effect, which increases the material’s overall *E* [7,26]. Ji et al. [60] confirmed that cement-based composite materials with higher elastic moduli are better able to resist deformation during FTs, preventing premature crack formation. Therefore, it is concluded that the combined effects of the fiber bridging effect and the hardening effect of the pore ice contribute to the increased initial cracking strength of ECC specimens in the FS.

#### 3.2.4. Ultimate Flexural Strength

**Figure 10:**
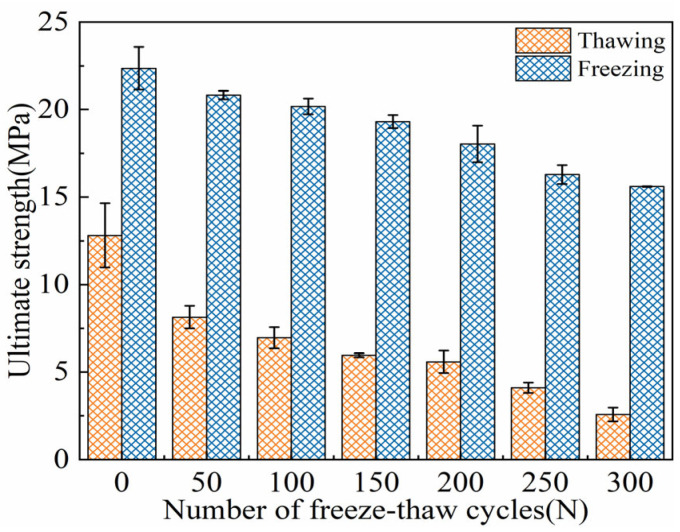
Freezing-thawing evolution characteristics of US of ECC specimens in UFS and FS.

As shown in Figure 10, the evolution of the ultimate strength of ECC specimens under FTs0–FTs300 in both TS and FS is presented. With the increase in FTs, the ultimate strength of ECC specimens in both states gradually decreases. At zero FTs, the ultimate strength in the TS and FS are 12.81 MPa and 23.74 MPa, respectively. After FTs300, the ultimate strength decreases to 2.58 MPa and 15.63 MPa, respectively. Throughout the FTs, the ultimate strength of the ECC specimens in the FS remains higher than that in the TS. The difference in ultimate strength between the TS and FS ranges from 10.93 MPa to 13.20 MPa, which is slightly lower than the difference in initial cracking strength between the two states. The analysis suggests that the increase in the ultimate flexural strength of ECC in the FS is attributed to the freezing of pore water at low temperatures, which fills the microcracks and pores in ECC, thereby enhancing the density of the matrix. Additionally, the bond strength of the fibers in the ECC matrix increases [7,26,49]. As a result, the initial cracking strength and ultimate flexural strength of ECCs in the FS are significantly higher than those in the TS.

The bending strength gain ratio γf0→γf300 is as follows: 74%, 156%, 190%, 224%, 223%, 298%, and 505%. As the FTs increase, the strength gain ratio at low temperatures gradually increases. Over the entire cycle, the increase is characterized by a slow rise during the early freeze–thaw period (FTs0–FTs100), a relatively flat development in the middle period (FTs100–FTs200), and a rapid increase in the later period (FTs200–FTs300). Before the FTs, the gain ratio γf0 is 74%, and after 300 cycles, the gain ratio γf300 increases to 505%. The analysis suggests that the gain ratio is primarily caused by the freeze–thaw expansion force generated by the phase change of pore water into ice. The freeze–thaw expansion provides a form of “prestress” for the specimen [7]. When the FTD is significant, the strength degradation of the specimen in the TS is severe. However, in the case of significant FTD, the contribution of “prestress” to the load-bearing capacity of the specimen in the FS becomes increasingly prominent, ultimately leading to the continuous increase in the gain ratio.

In addition, under the TS, the ultimate strength of ECC specimens in each FT is significantly higher than their cracking strength. However, in the FS, the difference between ultimate strength and cracking strength is not substantial. This phenomenon can be attributed to the fact that the cracking strength of ECCs mainly depends on the matrix strength of the cement-based material and its internal microstructural characteristics, while the peak flexural strength of ECCs is closely related to both the matrix properties and the bonding performance at the fiber–matrix interface [49,56,61]. In the FS, the pore water within the ECC transforms into ice with certain rigidity, and the unhydrated cement particles and fine aggregates also freeze and harden, thereby enhancing the initial cracking flexural strength of ECCs. The phase change of pore water increases the bonding force between PVA fibers and the ECC matrix, improves the interfacial transition zone (ITZ) between the fibers and matrix, and enhances the bridging effect of the PVA fibers, which collectively contribute to the increase in the peak flexural strength of ECC [59]. However, the low-temperature effects may degrade the performance of the cementitious matrix, particularly the C–S–H gel. Additionally, the internal stress redistribution caused by the freezing of pore water, as well as stress concentration at microcrack tips in the matrix and especially at the ITZ between the matrix and fibers, makes this region highly susceptible to fracture [62]. This accelerates microcrack propagation and leads to pronounced brittleness at the macro level [49,62]. The failure modes observed during loading further illustrate this phenomenon. Under the TS, ECC specimens exhibit cracks that gradually develop until the ultimate load is reached, after which the specimens fail. In contrast, under the FS, ECC specimens fracture rapidly along the cracks shortly after reaching the cracking strength, which almost coincides with the ultimate strength. The freeze–thaw evolution characteristics of flexural performance align with the ductile failure characteristics in the TS and brittle failure characteristics in the FS.

#### 3.2.5. Ultimate Peak Deflection

**Figure 11:**
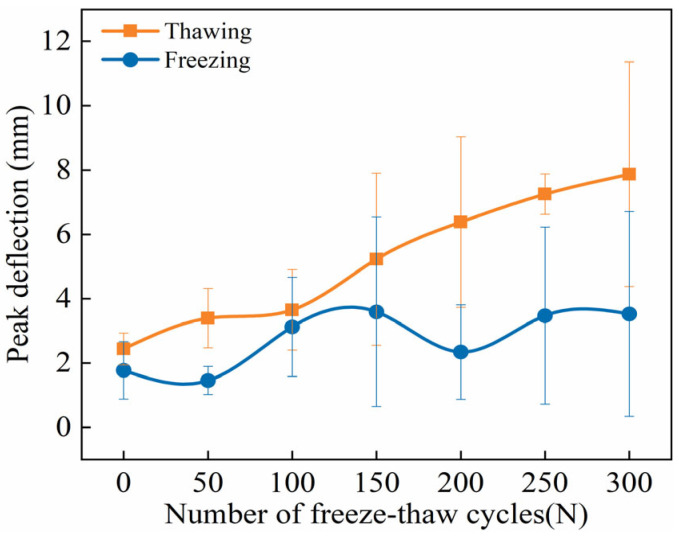
FTE characteristics of peak deflection of ECC specimens under UFS and FS.

As shown in Figure 11, the evolution of the ultimate peak deflection of ECC specimens during FTs0–FTs300 under both TS and FS is presented. It can be observed that with the increase in FTs, the overall ultimate peak deflection in both states exhibits an increasing trend. By comparing the two curves, it is evident that at each FT, the ultimate peak deflection in the TS consistently exceeds that in the FS. Moreover, as the number of FTs increases, the deflection difference between the two states gradually widens.

The analysis suggests that as FTs progress, the bending deflection of ECC specimens in the TS increases continuously due to the increased number of cracks and fiber pull-outs in the flexural-tensile section. In contrast, in the low-temperature FS, the freezing of internal pore water reduces material porosity, mitigates the effects of initial defects, and enhances material strength and *E*. Additionally, the volumetric expansion caused by ice formation increases the gripping force of the matrix on the fibers. These factors strengthen the bond between the fibers and the matrix, thereby reducing the irreversible pull-out and slippage of fibers at both ends [49,60,62]. When ECC specimens under the FS are subjected to bending loads, the presence of ice bridges microcracks, enhancing the interface among fibers, aggregates, and the cementitious matrix. Initially, the microcracks are restrained by the fiber-ice-matrix composite zone, which hinders crack propagation. As the bending load increases, once a crack penetrates the fiber–ice–matrix composite zone, the aggregate–ice–matrix composite zone further prevents crack expansion. These two inhibition mechanisms alternate during the gradual crack propagation process until the main crack resistance within ECCs weakens, leading to the onset of unstable crack growth [7,62]. Therefore, ECCs exhibit significant brittleness at low temperatures, with FTD contributing minimally to toughness. This explains the observed trend of an increasing deflection difference between the TS and FS over FTs.

#### 3.2.6. Flexural Toughness Index

Flexural toughness is crucial for ECCs as a high-ductility cement-based materials. While the ultimate peak deflection *δ_fu_* to some extent reflects the flexural deformation capacity of ECC specimens, relying solely on *δ_fu_* cannot fully assess ECCs’ flexural toughness, and the variation in *δ_fu_* does not exhibit a clear pattern. Drawing from energy considerations, following references [63,64], the flexural toughness index *T_u/c_* is defined as the ratio of the ultimate peak fracture energy to the initial cracking energy. Subsequently, the evolution characteristics of ECC flexural toughness under FT conditions are described through the changes in *T_u/c_*.

**Figure 12:**
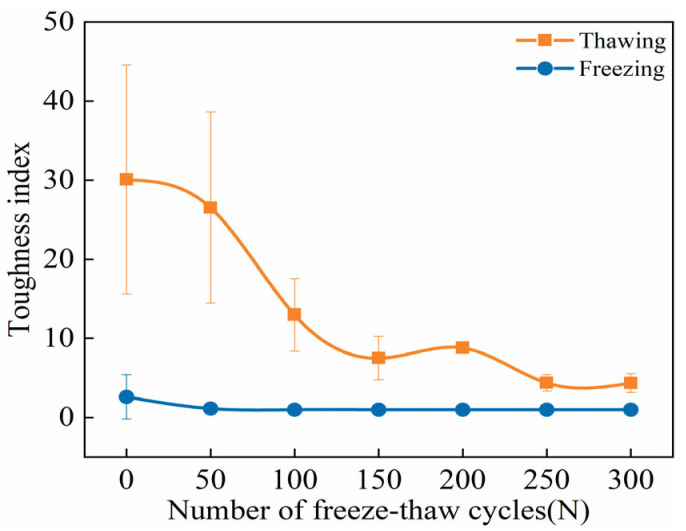
FTE characteristics of flexural toughness index of ECC specimens under UFS and FS.

As shown in Figure 12, the evolution of the flexural toughness index *T_u/c_* of ECC specimens during FTs0–FTs300 exhibits distinct differences between the TS and FS. It is evident from the graph that with the increase in FTs, the *T_u/c_* of ECC specimens in the TS exhibit an overall decreasing trend. Analysis suggests that there may be certain characteristic FT numbers *N_ci_* at which the trend of increase or decrease in the flexural toughness index *T_u/c_* changes. In the early stages of FTs, when N < *N_c_*_1_, *T_u/c_* gradually decreases. However, when the number of FTs exceeds *N_c_*_1_, *T_u/c_* starts to show a slight increasing trend. Upon reaching *N_c_*_2_ FTs, the trend in the increase of *T_u/c_* changes, stabilizing or fluctuating around a constant value. The characteristic FT numbers *N_c_*_1_ and *N_c_*_2_ are approximately 150 and 200, respectively. The experimental results indicate a relative dynamic equilibrium between ECC strength and deformation. For toughness, neither an absolute decrease in strength nor an absolute increase in deformation necessarily implies an improvement in toughness. Studies have shown that a relative balance between ECC strength and deformability is required [65]. Therefore, the characteristic FT numbers *N_ci_* are indeed real, and they should be noticed when assessing the changes in the flexural toughness index *T_u/c_* during the FT process.

In the FS, the flexural toughness index is only observed before FTs begin, with the index dropping to 1 during the FTs50–FTs300 cycle range, indicating no theoretical toughness. The flexural toughness index is defined as the ratio of the area under the ultimate peak strength curve to the area under the initial cracking strength curve. Based on the load-displacement curves in the FS, it is evident that ECCs undergo a sudden load drop during loading, displaying pronounced brittle failure characteristics. After FTs50, the initial cracking point coincides with the ultimate peak point, making the initial crack the ultimate strength. Additionally, freezing temperatures cause PVA fibers, the cement matrix, pore water, unhydrated cement particles, and fine aggregates to harden, depriving ECCs of their high energy absorption efficiency and elastic properties. This results in increased rigidity and brittleness, reducing the flexural strength absorption of ECCs and further increasing their brittleness. Moreover, due to the differences in thermal expansion coefficients among the components within ECCs, interface stress is induced at low temperatures [57]. This accelerates the propagation of microcracks in weak areas of ECCs, ultimately leading to brittle failure. Therefore, the theoretical flexural toughness index in the FS remains 1 throughout the FTs, consistent with its quasi-brittle failure mode and findings reported in the literature [56,57,61].

## 4. Degradation Mechanism of MP of ECC Under Freezing Conditions

According to the experimental results, both the strength and *E* of ECCs in the thawed and FS gradually decrease with the increase in FTs. This is due to the presence of a certain amount of free water within the cementitious matrix of ECCs, which does not participate in the hydration process. During the cooling process, this free water condenses into ice, filling the pores within ECCs and generating crystallization pressure, accompanied by volume expansion. At the same time, part of the water is pushed into nearby pores [7,62]. The growth of the ice increases the stress on the pore walls, which induces the formation of microcracks, resulting in the concrete exhibiting expansive behavior. When the temperature rises from low to ambient conditions, the ice in the pores gradually melts into water, and the internal structure returns to a looser state. The stress between the ice and the pore walls gradually decreases, leading to the shrinkage of the cementitious matrix, which further induces the formation of microcracks. Additionally, the freeze pressure causes the destruction of the pore structure, leading to a reduction in the strength of the ECC [49,51,62].

In the thawed state (TS), the strength of ECCs deteriorates significantly, whereas in the FS, the strength loss is much smaller. After FTs300, the peak strength of ECCs in the low-temperature FS remains around 20 MPa, and the ultimate flexural strength is still approximately 16 MPa. It is analyzed that in the FS, moisture within the ECC specimen condenses into ice, filling the internal pores. During the cooling process, the water in larger pores condenses first. In smaller pores, due to surface tension on the pore walls, the freezing point of the water decreases as the pore diameter decreases, leading to an increasing amount of water turning into ice inside the pores, which gradually increases the strength of the material. On the other hand, under low-temperature conditions, the bonding strength between the fibers and the matrix increases. During the fracture and deformation process, the energy absorbed by fiber pullout increases, which not only restricts crack slip but also bears the shear stress from external loads. As a result, only a few through cracks are formed in the ECC, further hindering its failure, and thereby increasing the CS, with a “crack but not break” failure mode [62,66]. Therefore, the prestress-bearing capacity provided by the pore ice and the bridging effect of the fibers prevent the rapid degradation of the ECC’s CS, and this phenomenon becomes more pronounced with a higher number of FTs. Throughout different FTs, the strength and *E* of ECCs in the FS are higher than in the TS, with the peak strength gain showing an overall increasing trend.

Considering the freeze–thaw evolution characteristics of ECC strength and *E*, it is found that as the FT number increases, the *E* of ECCs gradually decreases. When the number of FTs reaches a certain critical value, the *E* of ECCs begins to be lower than that of pore ice. At high FT numbers, the *E* of a specimen is mainly provided by the pore ice [7,26]. Therefore, when FTD is significant, the strength degradation of ECCs in the TS will be severe. However, in a frozen environment, the “prestress” provided by pore ice plays a very significant role in supporting the load in the FS. The hardening effect of the pore ice dominates, and the material damage caused by the pore ice is a secondary factor. This leads to an overall increase in ECC stiffness, a higher initial *E*, and improved durability. Thus, the actual *E* of ECCs in the FS differs greatly from that in the TS due to the presence of pore ice, resulting in the peak strength of ECCs in the FS being higher than that in the TS.

## 5. Durability Evaluation Model for ECCs

In previous research, the durability assessment of cement-based materials often relied on a singular indicator. For example, the American Society for Testing and Materials (ASTM) [67] utilizes the relative modulus of elasticity to gauge FT resistance. At the same time, in China, the evaluation involves either the relative modulus of elasticity or the mass loss rate [44]. However, following FTs, ECCs exhibit diverse changes in MPs in UFS or FS across various performance indicators. Consequently, accurately evaluating ECC durability in different states proves challenging with a single index.

Drawing from extensive prior research on ECCs’ compressive properties (CPs) conducted by our research group [7,26], this study integrates uniaxial compression test data of ECC specimens in UFS and FS. Concurrently, results from FP tests of ECC specimens conducted in both UFS and FS contribute to establishing a more comprehensive durability assessment model.

### 5.1. Durability Evaluation Model Is Established

CS is often used as a standard to evaluate cement-based materials durability in cement-based materials durability testing [68]. However, in this experiment, specimens’ UC strength, *E*, ICS, and flexural US vary under different FTs. Moreover, ECC specimens exhibit different MPs in both thawing and freezing test environments. Therefore, evaluating the durability of ECC materials comprehensively by considering all these MPs aligns better with engineering practice. Based on this, this paper establishes a comprehensive evaluation system for assessing the influence of FTs on the durability of ECC materials in both thawing and freezing test environments. The detailed derivation process of the evaluation model is provided in the Supplementary File “Derivation process of ECCs durability evaluation model”. For the weighting of different indicators, the entropy weight method is adopted [68], as detailed below: To establish a comprehensive durability evaluation index *S* for ECCs, the normalized durability index value *S*_*i*_ and weight *t*_*i*_ are used. The evaluation matrix is constructed using the uniaxial CS, *E*, initial crack strength, and ultimate strength of the specimens $A={\left(a_{ij}\right)}_{mn}$$\left(i=1,2,\ldots ,m;j=1,2,\ldots ,n\right)$. Here, *n* represents the number of evaluation objects, and *m* represents the number of evaluation indices. The data in the matrix are first standardized to obtain a new judgment matrix, and then the weight (*P*), entropy value (*e*), and the weight of each index (*W*) for the *j*-th index of the *i*-th sample are calculated. Finally, the durability value (*D*) for each sample is computed using the following formula:

(3) $$D_{i}=\sum_{j=1}^{n}X_{ij}W_{ij}$$

### 5.2. Predicting the Service Life of ECCs Development of Life Prediction Model

Generally, a structure’s SL or durability refers to the time the system can fulfill its designated function under normal usage and maintenance conditions. As construction materials, ECCs’ SL is intricately linked to the lifespan of the entire building. Therefore, evaluating the SL of ECCs is a crucial task. In cold regions, ECC deterioration is induced by the damage to its inherent structure. The degradation process signifies the progression of self-structural damage. The degradation variable corresponds to the extent of damage [68]. Let *X*_0_ denote the original quantity of ECCs (such as the value of durability *D*), *X_t_* represents the remaining undamaged quantity of ECCs up to a certain point of failure, and *λ* is the natural decay constant. Thus, the decay equation is expressed as follows:

(4) $$\frac{dX_{t}}{d_{t}}=-\lambda (X_{t}-X_{0})$$

(5) $$\frac{X_{t}}{X_{0}}=e^{-\lambda t}$$

The decay equation for cement-based materials closely approximates the cooling law of Newtonian matter [68]. Hence, the GM (1,1) grey system theory model can reasonably predict the durability (*D*) values for ECC specimens subjected to FTs in UFS and FS.

Let $\left\{X^{\left(0\right)}(k)|x^{\left(0\right)}\left(1\right),x^{\left(0\right)}\left(2\right),\ldots ,x^{\left(0\right)}\left(n\right)\right\}$ represent the original sequence of durability values *D* for ECC specimens in both TS and FS, and let $\left\{X^{\left(1\right)}(k)|x^{\left(1\right)}(1),x^{\left(1\right)}(2),\ldots ,x^{\left(1\right)}(n)\right\}$ and $Z^{\left(1\right)}$ be the 1-AGO sequence and the nearest mean generated sequence of $X^{\left(1\right)}$, respectively. Then, a first-order differential equation model for the variables is established with equal spacing.

(6) $$\frac{dX^{\left(1\right)}}{dk}+aX^{\left(1\right)}=u$$

where *a* and *u* are undetermined parameters within the equation.

The newly generated sequence obtained by accumulating the original sequence should satisfy the following functional relationship:

(7) $$x^{\left(1\right)}(k)=\left[x^{\left(0\right)}(1)-\frac{u}{a}\right]e^{-a\left(k-1\right)}+\frac{u}{a}(k=1,2,3,\ldots ,n)$$

Construct the parameter matrix $\overset{\wedge }{a}$

(8) $$\overset{\wedge }{a}={\left[a,u\right]}^{T}$$

The parameter matrix $\overset{\wedge }{a}$ adheres to the relation

(9) $$\overset{\wedge }{a}={\left(B^{T}B\right)}^{-1}B^{T}Y$$

where [*B*] and [*Y*] are

(10) $$B=\left[\begin{array}{ll} Z^{\left(1\right)}(2) & 1\\ Z^{\left(1\right)}(3) & 1\\ \;\;\;\ldots & \ldots \\ Z^{\left(1\right)}(n) & 1 \end{array}\right]=\left[\begin{array}{ll} -\frac{1}{2}(x^{\left(1\right)}(1)+x^{\left(1\right)}(2)) & 1\\ -\frac{1}{2}(x^{\left(1\right)}(2)+x^{\left(1\right)}(3)) & 1\\ \;\;\;\;\;\;\;\;\;\;\;\;\;\;\;\;\;\;\;\;\;\ldots & \ldots \\ -\frac{1}{2}(x^{\left(1\right)}(n-1)+x^{\left(1\right)}(n)) & 1 \end{array}\right]$$

(11) $$Y={\left[x^{\left(0\right)}(2),x^{\left(0\right)}(3),\ldots ,x^{\left(0\right)}(n)\right]}^{T}$$

The corresponding functions for the differential equation are

(12) $$x^{\prime (0)}\left(k\right)=x^{\left(1\right)}\left(k\right)-x^{\left(1\right)}(k-1)\left(k=1,2,\ldots ,n\right)$$

where $x^{\prime (0)}\left(k\right)$ represents the predicted value for the kth item.

The time response equation for $x^{\prime (0)}\left(k\right)$ is then

(13) $$x^{\prime (0)}\left(k\right)=\left(1-e^{a}\right)\left(x^{\left(0\right)}(1)-\frac{u}{a}\right)e^{-a(k-1)},k=1,2,\ldots ,n$$

To ensure the grey model’s accuracy, it should be subject to rigorous testing. We assess the established model through the post-error ratio method and the small probability error test. Table 9 outlines the criteria for the post-error ratio method.

In this table, we calculate $c=\frac{\sigma_{2}}{\sigma_{1}}$ as follows, with $\sigma_{1}$ and $\sigma_{2}$ denoting the standard deviations of the original strength data sequence and the residual sequence (the disparity between experimental values and predicted values)

(14) $$\bar{x^{\left(0\right)}}=\frac{1}{n}\sum_{i=1}^{n}x^{\left(0\right)}(k)$$

(15) $$\sigma_{1}^{2}=\frac{1}{n}{\sum_{i=1}^{n}\left[x^{\left(0\right)}(k)-\bar{x^{\left(0\right)}}\right]}^{2}$$

(16) $$q^{\left(0\right)}(k)=x^{\left(0\right)}(k)-x^{\prime (0)}(k)$$

(17) $$\bar{q}=\frac{1}{n-1}\sum_{i=1}^{n-1}q^{\left(0\right)}(k)$$

(18) $$\sigma_{2}^{2}=\frac{1}{n-1}{\sum_{i=1}^{n-1}\left[q^{\left(0\right)}(k)-\bar{q}\right]}^{2}$$

The computation for the small probability error *P* is as follows:

(19) $$P=P\left(|q^{\left(0\right)}(k)-\bar{q}|<0.6745\sigma_{1}\right)$$

### 5.3. Evaluation of the Durability of ECCs

Table 10 provides the impact weights of the four indicators on ECC specimen durability in both UFS and FS. Notably, in the TS, UC strength exerts the highest weight at 0.3644, followed by the *E* at 0.3376. This indicates that, under these conditions, ECC specimen durability is primarily influenced by their UC performance. In contrast, the FS presents a different scenario, where the weight of the *E* significantly increases to 0.6926, while the weight of UC strength decreases to 0.2257. As previously mentioned, pore ice in sub-zero temperatures triggers a hardening effect in ECC specimens, resulting in an enhanced *E* and increased specimen strength. This phenomenon aligns with the observed influence of *E* on specimen durability in FS, as discussed in prior research findings.

**Figure 13:**
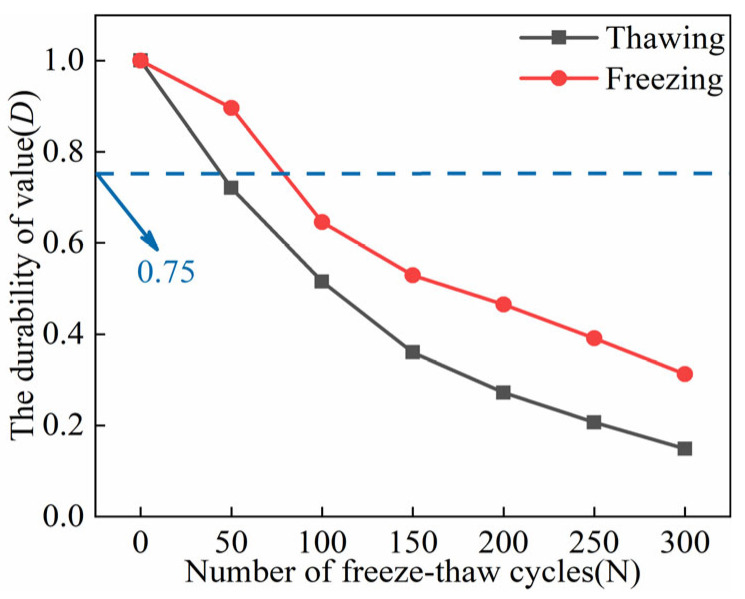
Durability values (*D*) of ECC specimens in UFS and FS (FTs0–FTs300).

Figure 13 depicts the durability values of ECC specimens in two different environmental states across various FTs. Overall, ECC specimens in both UFS and FS exhibit increasing damage with an increase in FTs. During FTs0–FTs50, the slope of the durability curve for ECC specimens in the FS is notably lower than that of specimens in the UFS. This suggests that pore ice in sub-zero temperatures enhances the *E* and stiffness of ECC specimens, making them more resistant to FTD. In FTs50–FTs100, ECC specimens in the FS experience a faster damage rate than those in the UFS. After FTs300, the durability value for ECC in the UFS decreases from 0.7207–0.1487, representing a loss of 79.37%. For ECC in the FS, the durability value decreases from 0.8960–0.3125, with a loss rate of 65.12%, significantly lower than that of UFS specimens. It is worth noting that concrete specimens are typically considered damaged when their CS falls below 75% [44]. Therefore, considering a durability value of *D* < 0.75 as a criterion for ECC specimen failure, ECC specimens in the UFS fail within the FTs0–FTs50 cycle range. In contrast, ECC specimens in the FS fail within the FTs50–FTs100 cycle range. Comparing the results of UC strength tests with the durability value *D*, it can be observed that the range of FTs required for *D* < 0.75 aligns with the range required when UC strength falls below the 75% benchmark value. This suggests that the established ECC durability assessment model has practical relevance and accuracy. However, due to the span of FTs in the experimental design, which does not entirely cover the specific periods of damage and failure for ECC specimens in UFS and FS, further research and modeling are needed for accurately predicting the lifetimes of ECC specimens in these conditions.

### 5.4. Predicting the Service Life of ECC

Table 11 presents the time response equations and model parameters to predict the durability values (*D*). These equations are employed to calculate the predicted SL values for ECC specimens in both UFS and FS, as detailed in Table 12. The table illustrates that both sets of predicted values fall within the range of experimental values, indicating the reasonable accuracy and applicability of the established SL prediction model. By combining the experimental and model-predicted values, it can be observed that ECC specimens subjected to FTD in the UFS will reach failure at FTs41, while in the FS, specimens will fail at FTs81.

Table 13 presents the outcomes of the model error tests. All post-error ratios (*C*) are less than 0.35, and all minor probability errors (*P*) are more significant than 0.80. These test results meet the requirements, indicating that the established model’s accuracy is satisfactory. It can predict the SL of FTD ECC specimens in both UFS and FS.

## 6. Conclusions

In a cold environment, experimental studies were conducted on the MPs of ECC specimens, and an LPM was proposed based on the test results. The following conclusions were drawn:

(1) In the FS, the pore water in ECC specimens turns to ice, providing “prestress”. As the number of FTs increases, the *E* of ECCs gradually decreases. When the number of FTs reaches a critical value, the *E* of the ECC becomes less than that of the pore ice, and the *E* of the specimens is primarily provided by the pore ice.

(2) ECC specimens under FS exhibited complete fracture into two parts during flexural failure, with relatively smooth fracture surfaces and fibers uniformly pulled apart internally, showing distinct brittle failure characteristics compared to specimens in TS. Furthermore, the development of the load-deflection curve for ECC specimens under FS showed significant variation compared to TS, with a severe degradation, or even disappearance, of deformation hardening behavior.

(3) Across different FTs, the initial cracking strength and ultimate peak FS under FS were significantly higher than in TS, with the ultimate peak deflection in FS being smaller than in TS. Throughout FTs0–FTs300, the FS gain ratio increase was consistent with CS. The flexural toughness index under FS was only evident before freezing and thawing; within FTs50–FTs300, there was theoretically no flexural toughness.

(4) LPMs for ECC specimens under TS and FS were established based on the calculated FT durability values. The predicted life values of each group of specimens fell within the experimental range, validating the reasonableness of the prediction results. The accuracy of the LPM met the requirements, providing a theoretical reference for predicting the SL of ECCs subjected to FTD in cold environments.

## Figures and Tables

**Table 1:** Composition of Portland cement oxides.

SiO_2_	Al_2_O_3_	CaO	Fe_2_O_3_	MgO	SO_2_	Ignition Loss
23.44	7.19	55.01	2.96	2.24	2.87	2.86

**Table 2:** Physical properties of Portland cement.

Setting Time (h)		Water Requirement of Normal Consistency (%)	FS (MPa)		CS (MPa)	
Initial	Final		3d	28d	3d	28d
1.95	2.98	26.93	5.82	8.14	28.92	47.64

**Table 3:** Oxide composition of FA.

SiO_2_	Al_2_O_3_	CaO	Fe_2_O_3_	MgO	K_2_O	Na_2_O	Others
40.28	18.15	18.08	8.56	2.34	1.76	1.31	2.26

**Table 4:** Main indexes of quartz sand.

Title	Size Range (mm)	SiO_2_	Fe_2_O_3_ (<%)	Moisture (<%)	Refractoriness (°C)
Silica sand	0.10–0.20	98.5	0.05	0.1Moisture	1750

**Table 5:** Performance index of PVA fiber.

Spcifications	Fineness (dtex)	Density (g.cm^3^)	Diameter (μm)	Length (mm)	*E* (GPa)	Elongation (%)	Tensile Strength (MPa)
K II	15	1.3	40	12	40	6	1600

**Table 6:** Mix proportions of raw materials of ECC (kg.m^−3^).

Cement	FA	Silica Sand	Water	Superplasticizer	*m*_w_/*m*_b_	PVA Fiber (vol%)
252.64	1010.57	454.76	303.17	17.68	0.24	2.00

Note: *m*_w_ represents water consumption; *m*_b_ represents cementitious material consumption; *m*_w_/*m*_b_ represents water binder ratio.

**Table 7:** Uniaxial compression performance parameters of ECC samples under thawing and freezing conditions [7,26].

FTs	Thawing			Freezing			Gain Ratio (%)
	*f*_cp_ (MPa)	*E* (GPa)	*ε*_cp_ (%)	*f*_cp_ (MPa)	*E* (GPa)	*ε*_cp_ (%)	
0	40.80	28.30	1.56	44.68	34.26	2.01	9.50
50	29.76	22.42	1.39	36.89	31.30	2.52	23.97
100	21.70	13.66	1.34	31.10	20.47	3.04	43.34
150	11.40	9.73	1.91	27.61	15.84	2.77	142.31
200	7.85	6.87	2.42	24.77	13.52	2.94	215.48
250	6.28	4.17	2.43	22.15	10.84	2.71	252.62
300	5.10	3.61	2.48	18.97	7.87	3.49	272.28

**Table 8:** Flexural MP parameters of ECC specimens in UFS and FS.

FTs	Thawing				Freezing				Gain Ratio (%)
	*f_f_*_c_ (MPa)	*f_f_*_u_ (MPa)	δ*_f_*_u_ (mm)	*T*_u/c_	*f_f_*_c_ (MPa)	*f_f_*_u_ (MPa)	δ*_f_*_u_ (mm)	*T*_u/c_	
0	7.12	12.81	2.44	30.07	21.65	22.35	1.77	2.62	74
50	4.44	8.13	3.40	26.52	20.52	20.82	1.46	1.15	156
100	3.71	6.96	3.65	12.97	20.71	20.17	3.12	1.00	190
150	3.50	5.96	5.23	7.49	18.04	19.30	3.59	1.00	224
200	2.62	5.58	6.38	8.81	18.04	18.04	2.34	1.00	223
250	2.55	4.09	7.25	4.36	16.19	16.29	3.47	1.00	298
300	1.44	2.58	7.87	4.34	15.43	15.60	3.53	1.00	505

**Table 9:** Posterior difference ratio test criteria.

Prediction Accuracy Level	c
Level 1: Good	<0.35
Level 2: Qualified	<0.50
Level 3: Reluctant	<0.65
Level 4: Unqualified	≥0.65

**Table 10:** Index weight (W).

Group	CS	*E*	Initial Crack Strength	US
Thawing	0.3644	0.3376	0.1494	0.1485
Freezing	0.2257	0.6926	0.0424	0.0393

**Table 11:** Prediction model of the durability value (*D*).

Group	*a*	*u*	*D*-Value Prediction Equation
Thawing	0.3190	1.1473	0.9756*e*^−0.3190(*k*−1)^
Freezing	0.2093	1.1537	1.0506*e*^−0.2093(*k*−1)^

**Table 12:** Cycle when the durability value (*D*) drops to 0.75.

Group	Predicted Value/(N)	Experimental Value/(N)
Thawing	41	0–50
Freezing	81	50–100

**Table 13:** The test results of model error.

Group	*c*	*p*
Thawing	0.03	1
Freezing	0.12	1

## Data Availability

The data that support the findings of this study are available from the corresponding authors on reasonable request due to privacy.

## Supplementary Materials

The following supporting information can be downloaded at: https://www.mdpi.com/article/10.3390/ma18102375/s1. Supporting File: Derivation process of ECCs durability evaluation model.
