# Supplementary material for: Characterization of Mechanical Property Evolution and Durability Life Prediction of Engineered Cementitious Composites Under Frozen State
Source: Materials (Basel). 2025 May 20;18(10):2375. doi: 10.3390/ma18102375 (PMC12113043; doi:10.3390/ma18102375)
Supplement: Supplementary file 1 [file materials-18-02375-s001.zip › materials-3415828-supplementary.pdf]

Derivation process of ECCs durability evaluation model

CS is often used as a standard to evaluate cement-based materials' durability in cement-based material durability testing. However, in this experiment, specimens' UC strength, E, ICS, and flexural US vary under different FTs. Moreover, ECC specimens exhibit different MPs in both thawing and freezing test environments. Therefore, evaluating the durability of ECC materials comprehensively by considering all these MPs aligns better with engineering practice. Based on this, this paper establishes a comprehensive evaluation system for assessing the influence of FTs on the durability of ECC materials in both thawing and freezing test environments, referencing the principles of the entropy weight method to assign weights to various indicators [68], as detailed below:

$$S = r_1 S_1 + r_2 S_2 + \ldots + r_n S_n = \sum_{i=1}^{n} r_i S_i \tag{S1}$$

In the equation, r_i represents the weight of the i indicator, and S_i represents the standardized indicator.

Build judgment matrix $A=(a_{ij})_{mn}$ using the UC strength, E, ICS, and US of the specimens, where (i=1, 2, ..., m; j=1, 2, ..., n). In this context, m represents the number of objects being assessed, and n represents the evaluation criteria. Normalize the data sequences within the matrix to derive a new judgment matrix X=$(X_{ij})_{mn}$ (i=1, 2, ..., m; j=1, 2, ..., n), following the standardization method outlined below:

$$x_{ij} = \frac{a_{ij}}{a_{1j}} \tag{S2}$$

Compute the weight (X') of the j index within the i specimen as follows:

$$X'_{ij} = \frac{X_{ij}}{\sum_{i=1}^{m} X_{ij}}, i = 1,2,...,m; j = 1,2,...,n \tag{S3}$$

Determine the entropy value (Δ) for the j index using the following formula:

$$\Delta_j = -\frac{1}{\ln(m)} \sum_{i=1}^{m} X'_{ij} \ln\left(X'_{ij}\right), j = 1,2,...,n \tag{S4}$$

Calculate the weight (r) for each index with the following equation:

$$r_j = \frac{1-\Delta_j}{\sum_{j=1}^{n}\left(1-\Delta_j\right)}, j = 1,2,...,n \tag{S5}$$

Subsequently, obtain the durability value (D) for each specimen through the following formula:

$$D_i = \sum_{j=1}^{n} X_{ij} r_{ij} \tag{S6}$$

Generally, a structure's SL or durability refers to the time the system can fulfill its designated function under normal usage and maintenance conditions. As a construction material, the SL of ECCs is intricately linked to the lifespan of the entire building. Therefore, evaluating the SL of ECCs is a crucial task. In cold regions, ECC deterioration is induced by the damage to its inherent structure. The degradation process signifies the progression of self-structural damage. The degradation variable corresponds to the extent of damage[34]. Let X_0 denote the original quantity of ECCs (such as the value of durability D), X_t represents the remaining undamaged quantity of ECCs up to a certain point of failure, and λ is the natural decay constant. Thus, the decay equation is expressed as follows:

$$\frac{dX_t}{d_t} = -\lambda(X_t - X_0) \tag{S7}$$

$$\frac{X_t}{X_0} = e^{-\lambda t} \tag{S8}$$

The decay equation for cement-based materials closely approximates the cooling law of Newtonian matter[34]. Hence, the GM (1,1) grey system theory model can reasonably predict the durability (D) values for ECC specimens subjected to FTs in UFS and FS.

Let $\left\{X^{(0)}(k) \mid x^{(0)}(1), x^{(0)}(2), ..., x^{(0)}(n)\right\}$ represent the original numerical sequence of durability values (D) for PVA-ECC specimens in both UFS and FS. The corresponding 1-ago sequence, denoted as $\left\{X^{(1)}(k) \mid x^{(1)}(1), x^{(1)}(2), ..., x^{(1)}(n)\right\}$, can be defined as follows:

$$x^{(1)}(k) = \sum_{i=1}^{k} x^{(0)}(i), k = 1, 2, , ..., n \tag{S9}$$

$W^{(1)}$ represents the sequence generated by the adjacent mean values of $X^{(1)}$, where $W^{(1)} = (W^{(1)}(2), W^{(1)}(3), ..., W^{(1)}(n))$:

$$W^{(1)}(k) = -\frac{1}{2}\left[x^{(1)}(k) + x^{(1)}(k-1)\right], k = 2, 3, ..., n \tag{S10}$$

Establish a first-order differential equation model for variables with evenly spaced intervals as follows:

$$\frac{dX^{(1)}}{dk} + aX^{(1)} = u \tag{S11}$$

Where "a" and "u" are undetermined parameters within the equation.

$$x^{(1)}(k) = \left[x^{(0)}(1) - \frac{u}{a}\right]e^{-a(k-1)} + \frac{u}{a}(k = 1, 2, 3, ..., n) \tag{S12}$$

The newly generated sequence obtained by accumulating the original sequence should satisfy the following functional relationship:

$$\hat{a}=[a,u]^T \tag{S13}$$

Construct the parameter matrix $\hat{a}$ as follows:

$$\hat{a}=\left(B^T B\right)^{-1} B^T Y \tag{S14}$$

The parameter matrix $\hat{a}$ adheres to the following relation:

$$\hat{a}=\left(B^T B\right)^{-1} B^T Y \tag{S15}$$

Where [B] and [Y] are defined as follows:

$$B=\begin{bmatrix} W^{(1)}(2) & 1 \\ W^{(1)}(3) & 1 \\ \cdots & \cdots \\ W^{(1)}(n) & 1 \end{bmatrix}=\begin{bmatrix} -\frac{1}{2}(x^{(1)}(1)+x^{(1)}(2)) & 1 \\ -\frac{1}{2}(x^{(1)}(2)+x^{(1)}(3)) & 1 \\ \cdots & \cdots \\ -\frac{1}{2}(x^{(1)}(n-1)+x^{(1)}(n)) & 1 \end{bmatrix} \tag{S16}$$

$$Y=\left[x^{(0)}(2),x^{(0)}(3),...,x^{(0)}(n)\right]^T \tag{S17}$$

The corresponding functions for the differential equation are as follows:

$$x^{'(0)}\left(k\right)=x^{(1)}\left(k\right)-x^{(1)}(k-1)\left(k=1,2,...,n\right) \tag{S18}$$

Where $x^{'(0)}\left(k\right)$ represents the predicted value for the kth item.

The time response equation for $x^{'(0)}\left(k\right)$ is then the following:

$$x^{'(0)}\left(k\right)=\left(1-e^{a}\right)\left(x^{(0)}(1)-\frac{u}{a}\right)e^{-a(k-1)},k=1,2,...,n \tag{S19}$$

To ensure the grey model's accuracy, it must be subject to rigorous testing. We assess the established model through the post-error ratio method and the small probability error test.

In this table, we calculate $c=\frac{\sigma_2}{\sigma_1}$ as follows, with σ_1 and σ_2 denoting the standard deviations of the original strength data sequence and the residual sequence (the disparity between experimental values and predicted values):

$$\overline{x^{(0)}}=\frac{1}{n}\sum_{i=1}^{n}x^{(0)}(k) \tag{S20}$$

$$\sigma_1^2=\frac{1}{n}\sum_{i=1}^{n}\left[x^{(0)}(k)-\overline{x^{(0)}}\right]^2 \tag{S21}$$

$$q^{(0)}(k)=x^{(0)}(k)-x^{'(0)}(k) \tag{S22}$$

$$\bar{q}=\frac{1}{n-1}\sum_{i=1}^{n-1}q^{(0)}(k) \tag{S23}$$

$$\sigma_2^2=\frac{1}{n-1}\sum_{i=1}^{n-1}\left[q^{(0)}(k)-\bar{q}\right]^2 \tag{S24}$$

The computation for the small probability error P is as follows:

$$P = P\left(| q^{(0)}(k) - \overline{q} | < 0.6745\sigma_1 \right) \quad \text{(S25)}$$
